# Annotation and Expression of IDN2-like and FDM-like Genes in Sexual and Aposporous *Hypericum perforatum* L. accessions

**DOI:** 10.3390/plants8060158

**Published:** 2019-06-07

**Authors:** Andrea Basso, Gianni Barcaccia, Giulio Galla

**Affiliations:** Laboratory of Genetics and Genomics, DAFNAE, University of Padova, Campus of Agripolis, Viale dell’ Università, 1635020 Legnaro, Italy; andrea.basso@unipd.it (A.B.); gianni.barcaccia@unipd.it (G.B.)

**Keywords:** *Hypericum perforatum* L., aposporous apomixis, RdDM, IDN2, FDM, auxin, cytokinin

## Abstract

The protein IDN2, together with the highly similar interactors FDM1 and FDM2, is required for RNA-directed DNA methylation (RdDM) and siRNA production. Epigenetic regulation of gene expression is required to restrict cell fate determination in *A. thaliana* ovules. Recently, three transcripts sharing high similarity with the *A. thaliana* IDN2 and FDM1-2 were found to be differentially expressed in ovules of apomictic *Hypericum perforatum* L. accessions. To gain further insight into the expression and regulation of these genes in the context of apomixis, we investigated genomic, transcriptional and functional aspects of the gene family in this species. The *H. perforatum* genome encodes for two IDN2-like and 7 FDM-like genes. Differential and heterochronic expression of FDM4-like genes was found in *H. perforatum* pistils. The involvement of these genes in reproduction and seed development is consistent with the observed reduction of the seed set and high variability in seed size in *A. thaliana* IDN2 and FDM-like knockout lines. Differential expression of IDN2-like and FDM-like genes in *H. perforatum* was predicted to affect the network of potential interactions between these proteins. Furthermore, pistil transcript levels are modulated by cytokinin and auxin but the effect operated by the two hormones depends on the reproductive phenotype.

## 1. Introduction

Apomixis defines a few plant reproductive strategies which permit the inheritance of the maternal genome without genetic recombination and syngamy. This reproductive strategy is characterized by the differentiation of functional embryos, either from a somatic cell of the ovule nucellus or from an unreduced female gametophyte. The development of unreduced functional gametes from unorthodox cell precursors (i.e., different from the meiotically reduced functional megaspore) is a defining characteristic of a subset of reproductive strategies known as gametophytic apomixis (for reviews on apomixis, see References [1,2]). Among these strategies, the aposporous type of apomixis is characterized by the differentiation of functional embryo sacs (ESs) from somatic cells of the ovule. Histological observations in several aposporous species have indicated that competence for ES founder cell specification extends to the cellular layers surrounding the meiocytes (i.e., the nucellus and/or the chalaza), rather than being restricted to the meiocyte cell type [3,4,5,6,7,8]. Results have shown that epigenetic regulation of gene expression by either DNA methylation or posttranscriptional gene silencing (PTGS) is required for proper ovule development in the model species *Arabidopsis thaliana* [9]. Accordingly, the activity of the RNA-dependent DNA methylation (RdDM) pathway during reproduction is essential for gametophyte development in this species and loss-of-function mutants for genes involved in this pathway result in aberrant cell fate establishment within the ovule with phenotypes that are strikingly reminiscent of apomictic development [9,10].

DNA methylation of RdDM target loci is mediated by the co-occurrence and interaction of small interfering RNAs (siRNAs), long noncoding RNAs (lncRNAs) and core RNA interference (RNAi) proteins, including INVOLVED IN DE NOVO 2 (IDN2) and its highly similar interactors FACTOR OF DNA METHYLATION 1 (FDM1) and FACTOR OF DNA METHYLATION 2 (FDM2) [11,12,13]. IDN2 belongs to a small plant-specific gene family whose members are characterized by the presence of three major protein domains (Zf-XS, XS and XH) and a coiled coil, which were named after Arabidopsis SGS3 and its rice homolog X1 [14,15]. The XS domain is an RRM-like RNA-binding domain [11,12] that is required for the binding of dsRNA with 5′-overhangs. The Zf-XS domain is a small finger-like motif conjugated with one or more zinc ions and is usually paired with an XS domain recognizing a specific sequence of nucleic acids [14,16]. The XH domain, which is also found in SGS3, is required for the interaction of IDN2 with FDM1 and FDM2 [17]. The coiled-coil domain, which is located between XS and the XH, is essential for IDN2 homodimerization [17]. The interaction of IDN2 with FDM1 and FDM2 is thought to be required for RdDM [11,12] and to be downstream of siRNA production [13]. IDN2 and FDM1 have been shown to bind the 5′-overhangs of dsRNAs *in vitro and* FDM1 has been shown to bind unmethylated DNA *in vitro* [11,18,19].

According to the current model of the molecular function of IDN2-FDM1 at RdDM-targeted loci [20], IDN2 potentially binds to lncRNA by recognizing the 5′-overhang of a double-stranded RNA consisting of siRNA and lncRNA. Following the binding of dsRNAs, IDN2 is required for the association of the methyltransferase DOMAINS REARRANGED METHYLTRANSFERASE2 (DRM2) with the lncRNA at RdDM-targeted loci [20]. At the chromatin level, IDN2 interacts with the SWI/SNF chromatin-remodelling complex, which alters the nucleosome position and facilitates RdDM [21]. Although FDM3, FDM4 and FDM5 are also involved in RdDM, their functional mechanisms remain to be identified [22].

Gene expression studies performed on the aposporous apomictic medicinal plant *H. perforatum* have recently shown that several genes involved in the RdDM pathway are differentially expressed in pistils collected from aposporous plants [23,24]. Furthermore, two independent studies identified an orthologue of *A. thaliana* IDN2 as differentially expressed in *H. perforatum* pistils and microdissected ovules, suggesting involvement in developmental processes that define the aposporous apomictic reproductive strategy. However, little is known regarding the composition of the IDN2 and FDM-like gene family in *H. perforatum*. Furthermore, no information is currently available with respect to the gene expression, transcriptional regulation and protein-protein interaction potential of family members in this species. To gain further insight into the expression and regulation of *IDN2* and *FDM*-like genes in aposporous apomixis, we performed detailed bioinformatic and transcriptional investigations in sexually reproducing *A. thaliana* and *H. perforatum*. Bioinformatic investigations were performed to address the gene family composition, the conservation of characteristic protein domains and gene expression in premeiotic ovules. Furthermore, detailed spatial and temporal expression studies were performed to investigate the expression of genes in this family throughout pistil and gamete development. Finally, the transcriptional regulation of *IDN2/FDM1-5* genes was addressed by computational annotation of promoter sequences and gene expression studies coupled with hormonal treatments.

## 2. Results

The *A. thaliana* genome encodes a single *IDN2* gene and five potential or known interactors, currently annotated as *FDM1-5*. Their expression was detectable throughout the plant in *A. thaliana*, including the shoot apical meristems, flowers and siliques (Table S1, Figure S1). The clustering of the *ATIDN2* and *ATFDM1-5* genes based on their expression levels revealed similar patterns for *ATIDN2* and *ATFDM1/ATFDM2*, showing relatively high expression in the shoot apex, particularly during the transition from the vegetative to the inflorescence stage and in flowers at early developmental stages (Flower stage 9). Within flowers, their expression was higher at the earliest developmental timepoints. Among the different flower parts, high expression was found in the pistils (referred to as carpels, flower stages 12 and 15) and in the ovary (the component of the pistil that bears the ovules (Table S2, Figure S1). *ATFDM4* was the gene with the highest expression in shoot apical inflorescences, flowers at early developmental stages, carpels and ovaries. Despite the lower expression observed in anthers (referred to as stamens, flower stages 12 and 15), *ATIDN2* and *ATFDM1-5* transcripts were detectable during male gametogenesis, exhibiting an overall decreasing trend. Seeds and siliques were characterized by two alternative expression patterns. Hence, while the expression of IDN2 and FDM1-3 peaked at seed developmental stages 4 and 7, *ATFDM4* and *ATFDM5* displayed a decreasing trend of expression throughout seed development.

**Figure 1:**
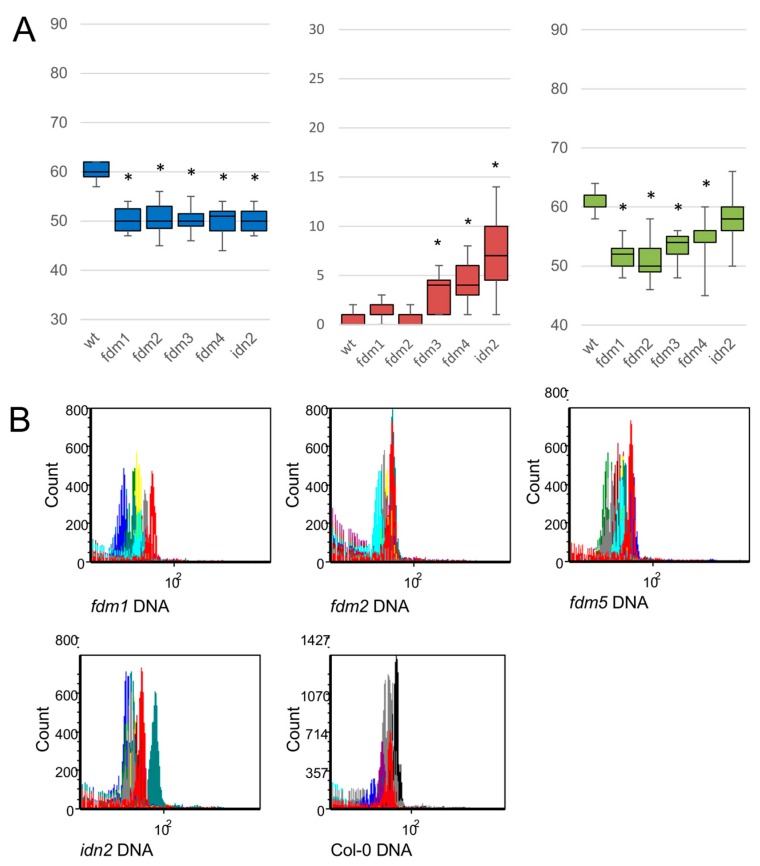
Seed set and flow cytometry screenings in *Arabidopsis* IDN2 and FDM-like knockout lines. (**A**): box plots reporting on the number of viable seeds/silique (blue boxes), the number of aborted seed/silique (red boxes) and the total number of seeds expressed as viable and aborted seeds/silique (green boxes). Boxes represent the 1st, 2nd and 3rd quartile, whiskers represent the observed minimum and maximum values. Significant differences (one-way ANOVA, *p*-value < 0.01) with WT Col-0 are reported as: *. (**B**): Flow cytometry screenings of single seeds. Each panel represent the overlay of 8 DNA histograms. The *X*-axis represent the fluorescent intensity of DAPI-staining and *Y*-axis the counts of measured nuclei.

To gain additional insights into *ATIDN2* and *ATFDM1-5* gene functions in reproductive processes, single-knockout mutants (Table S3) were obtained from Nottingham Arabidopsis Stock Centre (here after NASC) and investigated to determine the seed set (Figure 1), the average seed size (Table S4) and the embryo DNA content (Figure 1). *fdm5* seeds (SALK_075378) did not germinate in Kan- and Kan+ selective media. Additionally, several *idn2* and *fdm1-4* homozygous knockout plants (min: 3, max: 5) were selected, genotyped and analysed in detail (Figure S2). The mutant lines displayed a 15% reduction of the seed set (Figure 1A). While the number of aborted seeds in *fdm3*, *fdm4* and *idn2* plants was significantly higher than in the wild type (here after WT), the *fdm1* and *fdm2* lines were characterized by remarkably low abortion frequencies (Figure 1A, Figure S2). The mutation of single ATIDN2 and ATFDM1-4 genes is therefore considered to affect the plant seed set by affecting different developmental mechanisms. Notably, seed set variability was significantly higher in *fdm2*, *fdm4* and *idn2* than in WT accessions (Figure 1A). The seeds produced by the *idn2* and *fdm1-5* knockout lines were morphologically different from those of WT control plants (Table S2, Figure S2). More specifically, while the average seed area was significantly greater in the *idn2* and *fdm2* lines (respectively 0.137 ± 0.024 mm^2^ and 0.134 ± 0.019 mm^2^) than in the WT (0.126 ± 0.011 mm^2^), the remaining mutant lines produced smaller seeds than the WT (for more details, please refer to Table S2). The observed differences in seed size raised the question of whether embryo and/or endosperm DNA contents were affected by the knockout of these genes. The events assigned to endosperm cell populations in single seeds were numerically scarce and no significant differences were recorded among wild-type and mutant lines. However, *fdm1* (30%), *fdm5* (20%) and, to a lesser extent, *fdm2* (10%) and *idn2* (10%) displayed a significant reduction in the estimated embryo DNA content detected by flow cytometry screenings as count of bound from DAPI and embryo DNA (Figure 1B) [25].

### 2.1. Gene Annotation and Phylogenetic Analysis

To elucidate the composition and expression of *IDN2-like* and *FDM-like* genes in reproductive structures, we took advantage of recent sequencing efforts in *H. perforatum* sexual and aposporous apomictic accessions. *H. perforatum* aposporous apomicts are characterized by modifications in the ovule cell fate determination program, by which the competence differentiation of functional embryo sacs (ES) from somatic cells of the ovule instead of being restricted to a single reduced megaspore (i.e., the functional megaspore) deriving from meiosis [23,24,26,27]. Annotation of the sexual diploid genome of *H. perforatum* identified 9 genes encoding XH/XS-domain-containing proteins, sharing high sequence similarity with the *A. thaliana IDN2* and *FDM1-5* genes (Table 1).

**Figure 2:**
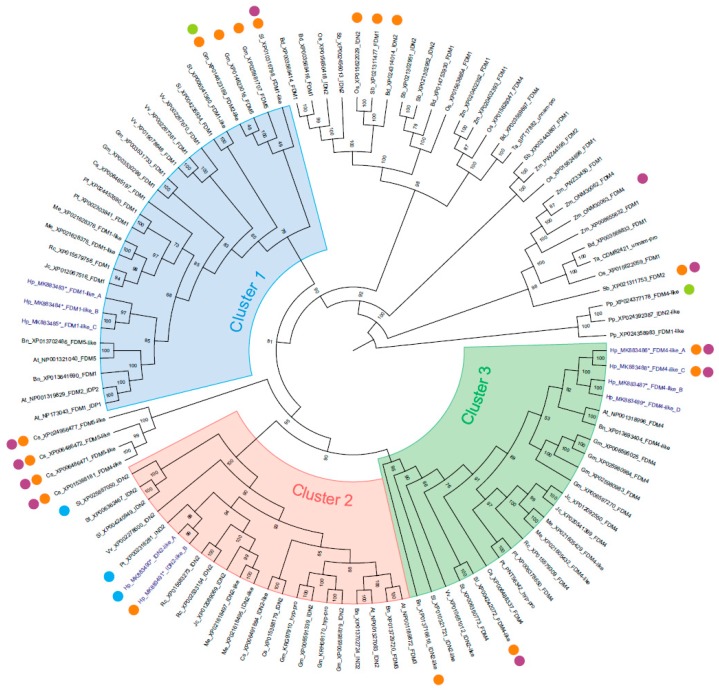
Maximum Likelihood tree (Log-likelihood: - 68069.81) from amino acids MAFFT alignments to investigate IDN2-like and FDM-like intra and inter-specific relationships. Ultra-fast bootstrap values are indicated on nodes. Coloured circles indicate differences in predicted protein domain composition. Orange: the Zf-XS domain was not found; violet: the XS domain was not found; green: the XH domain was not found; blue: multiple Zf-XS were predicted. The complete list of protein accessions considered in this study is reported on Table S5. Accession numbers marked with: * are referred to the gene sequences.

The annotated gene regions were 3385 bp in length on average, ranging from 2254 to 7304 bp. Two genes belonging to this family were predicted in a 30 kb sequence from ctg61981. Similarly, the contig sequence ctg61606 included four XH/XS domain-containing genes in a sequence window of approximately 20 kb (Table 1). Phylogenetic investigations performed on the available sequence data for 18 different species provided consistent clustering of the moss *Physcomitrella patens* (Pp), monocotyledon and dicotyledon protein sequences (Figure 2, Table S5). Within the dicotyledons, three main clusters (clusters 1 to 3) were detected. Cluster 1 (UFB support: 73) was defined by the presence of the *A. thaliana (At)* FDM1, FDM2 and FDM5 protein sequences, together with two *Populus trichocarpa* [28] proteins annotated as FDM1 [28]. Three *H. perforatum* sequences were grouped in this cluster, with UFB internode support of 97. This clade was sister to the clade that included sequences of *A. thaliana* and *Brassica napus* (Bn) and this relationship received robust statistical support (UFB= 85). According to this topology, the *H. perforatum* proteins included in this cluster were designated HPFDM1-like A, HPFDM1-like B and HPFDM1-like C. Cluster 2 included sequences annotated as IDN2 from *A. thaliana* and all other species considered in this investigation (Table S5). Two *H. perforatum* proteins were included in this cluster with very high bootstrap support. Cluster 3 grouped sequences annotated as FDM4 and IDN2 from the following species: *V. vinifera*, *B. napus* and *S. lycopersicum*. For *C. sinensis*, a unique cluster of protein sequences currently annotated as FDM4 and FDM5 was generated, situated between clusters one and two. For the *H. perforatum* sequences included in Cluster 3, the UFB support was high (UFB: 82). Accordingly, the *H. perforatum* sequences included in this group were designated HPFDM4-like A-D. It is worth noting that the *H. perforatum* FDM4-like sequences were divided into two subclusters with maximum support (i.e., UFB: 100). Estimates of the evolutionary divergence between the *H. perforatum* sequences (Table S3) were consistent with the clustering of *H. perforatum* sequences into three main clusters represented by the putative Arabidopsis orthologues *FDM1*, *FDM4* and IDN2.

### 2.2. Gene Expression Analysis

RNAseq gene expression analysis was focused on Laser Capture Microdissected (LCM) premeiotic ovules bulked from sexual and aposporous plant accessions. The sequencing data were analysed by using two complementary approaches. In the initial approach, the transcriptome assembled *de novo* from both sexual and apomictic sequence data was adopted as a reference (Table 1, Figure S3). The annotation of the transcriptome identified 18 *IDN2-like* and *FDM1,4-like* transcript variants. The alignment of the transcript variants to the annotated gene loci (Table 1) provided, on average, 2 variants per locus (min: 0, max: 4).

**Figure 3:**
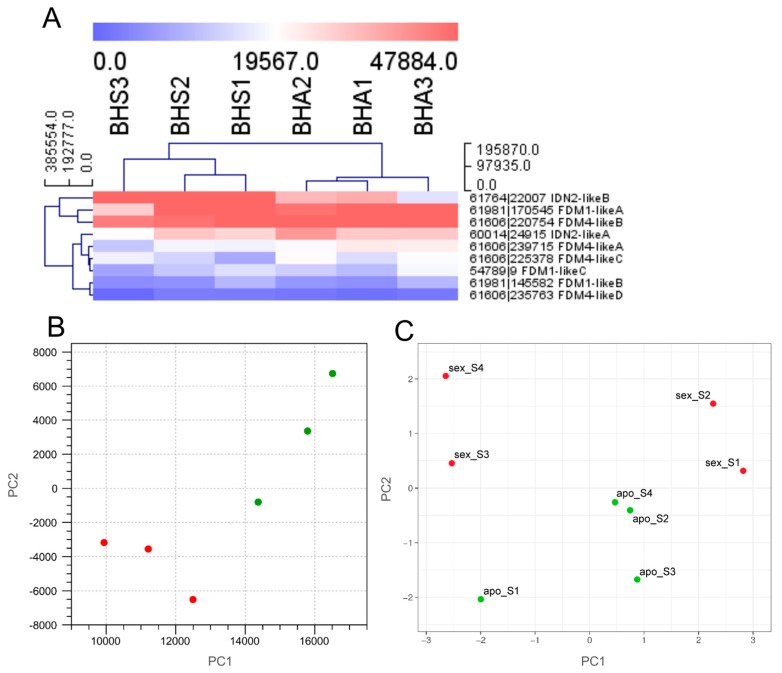
Expression of *H. perforatum* IDN2-like and FDM1,4-like genes. (**A**): Heat map showing the expression of IDN2-like and FDM1,4-like genes as assessed by RNAseq and by using the gene sequences predicted from the sexual diploid genome as reference. The HCL was performed by using Manhattan distances and average linkage clustering. BHS1-3: sexual samples; BHA1-3: aposporous apomictic samples. Gene loci are indicated as contig ID | start codon, followed by the gene name. Gene expression is reported as RPKM. Blue: low expression levels; Red: high expression levels. (**B**): Principal component analysis of IDN2-like and FDM1,4-like gene expression values in ovules, as assessed by RNAseq and by using the gene sequences predicted from the sexual diploid genome as reference. The percentage variation explained by the first and second component is 89% and 10%, respectively. Green circles: sexual samples; red circles: aposporous apomictic samples. (**C**): Principal component analysis of IDN2-like and FDM1,4-like gene expression values in ovules, as assessed by qPCR. The percentage variation explained by the first and second component is 51% and 22%, respectively. Green circles: sexual samples; red circles: aposporous apomictic samples. For both sexual and aposporous apomictic pistils, the following developmental stages were considered: S1: pre-meiosis; S2: meiosis; S3-S4 early and late gametogenesis, respectively.

Single transcript variants associated with the three genes *IDN2-like A*, *FDM1-like C* and *FDM4-like C* were differentially expressed in ovules (FDR *p*-value ≤ 7.0 E-04, Table 1). Notably, the clustering of samples on the basis of ovule expression data was consistent with the sample phenotypes (Figure S3 indicating that intraphenotype expression variation at the level of gene family was lower than that observed between antagonistic phenotypes.) In the second approach, mapping and GE analyses were performed on gene features predicted from the genome sequence of a diploid sexual accession, thereby focusing the investigation on gene features rather than transcript variants (Figure 3). The adoption of these last references did not alter the separation of samples according to their phenotype (Figure 3A,B). At this analytical level, GE analysis of sequencing data provided additional evidence of the downregulation of IDN2-like B in the reproductive structures of aposporous samples (Table S4).

Although the expression differences recorded for FDM1-like C and FDM4-like A-C were not significant, higher mean expression values were recorded in aposporous ovules (Table S4). Notably, the alignment of transcript sequences assembled de novo from sexual and apomictic ovules to sexual (i.e., predicted from the sexual diploid genome sequence) gene sequences resulted in high variability of the fraction of aligned transcript sequences and identity within the alignments. Furthermore, considering all transcript variants matching the FDM1-like C and FDM4-like C gene sequences, we noted that differentially expressed transcript variants l2_t405120_835163 (FDR:1.0E-6) and l2_t484402_835163 (FDR 9.0E-8) displayed the shortest length of alignment with the corresponding gene sequences (41.7% and 61.0%, respectively).

### 2.3. Protein-Protein Interactions

**Figure 4:**
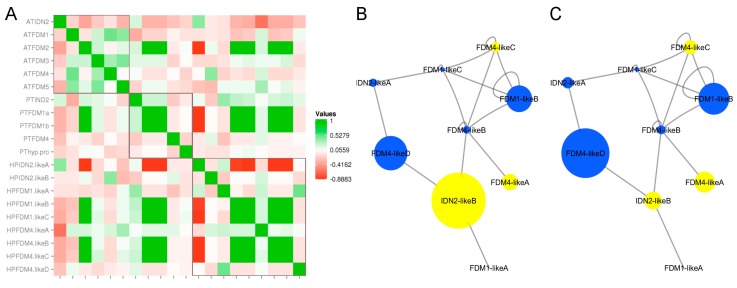
IDN2-like and FDM-like predicted protein-protein interactions. (**A**): Heat map showing Pearson correlation coefficients of *A. thaliana*, *P. trihocarpa* and *H. perforatum* PPIs scores. Negative PCC values are shown in red, while positive PCC values are shown in green. (**B**,**C**): networks representing predicted interactions among *H. perforatum* IDN2-like and FDM1,4-like proteins. Edges represent interactions predicted with PSOPIA (PPI score ≥ 0.419). Proteins lacking The XS and/or the Zf-XS domains are indicated as yellow nodes; Proteins displaying the XS and Zf-XS domains are indicated as blue nodes. Nodes size were set based on mean gene expression values in sexual and aposporous libraries (Table S4). B: PPI network was integrated with the mean gene expression values recorded in sexual ovules; C: PPI network was integrated with the mean gene expression values recorded in aposporous ovules.

Since interactions between *A. thaliana* IDN2 and FDM1 are expected to be required for RdDM [11,12] and to be downstream of small interfering RNA (siRNA) production [13], we tested the interaction potential of *H. perforatum* proteins by using sequence-based features. The domain architecture of IDN2-like and FDM1,4-like proteins was highly conserved in the investigated protein accessions (Figure 2, Table S5) and most *H. perforatum* proteins displayed the four characteristic domains: Zf-XS, XS, coiled-coil and HX domains (Figure 2). However, the two N-terminal domains involved in DNA/RNA binding (i.e., Zf-XS and XS) were not detected in the HPFDM4-like A and HPFDM4-like C sequences. Additionally, the Zf-XS domain but not the XS domain, was missing in HPIDN2-like B (Figure 2). Highly supported protein-protein interactions among the *H. perforatum* proteins were selected by using the experimentally determined value for the *A. thaliana* IDN2/FDM1 heterodimer (PPI score ≥ 0.419) as the cut-off value (Table 2). The Pearson correlation coefficients (PCCs) of the protein-protein interaction scores estimated for all pairwise comparisons between the predicted *A. thaliana, P. trichocarpa* and *H. perforatum* sequences are shown in Figure 4A.

Comparative analyses with the sexual model species *A. thaliana* and *P. trichocarpa* are relatively complex because of the increased number of protein sequences encoded by the *H. perforatum* genome. However, several potential interactions were conserved among species. For example, HPIDN2-like A and HPIDN2-like B displayed opposite interaction potentials with members of the HPFDM1-like clade (e.g., a low distance from HPFDM1-like A and a long distance from HPFDM1-like B and C), as observed for Arabidopsis FDM1, FDM2 and FDM5 (Figure 4A). Furthermore, the pattern of the interaction of Arabidopsis FDM4 with FDM5 was similar to those of HPFDM1-like A and two members of the FDM4-like clade (HPFDM4-like A and HPFDM4-like D). At the same time, the predicted interactions between protein pairs suggested some functional differentiation between members of the three protein subclades (i.e., HPIDN2, HPFDM1 and HPFDM4). More specifically, HPIDN2-like A and HPIDN2-like B displayed different interaction potentials with members of the HPFDM1-like and HPFDM4-like clades (Figure 4A, Table 2). The predicted interactors of HPFDM1-like B and HPFDM1-like C largely overlapped and differed from those predicted for HPFDM1-like A and the interactors predicted for HPFDM4-like B and HPFDM4-like C were different from those predicted for HPFDM4-like A and HPFDM4-like D (Figure 4A, Table 2). The integration of predicted PPIs with data related to the protein domain composition and ovule gene expression illustrated a complex network of potential interactions between HPIDN2-like and HPFDM1,4-like proteins (Figure 4B,C). Despite exhibiting relatively low expression levels (represented by the area of the node), FDM4-like B and FDM4-like C were characterized by the highest number of predicted interactions. As shown in Figure 4, proteins lacking one or both domains required for DNA and/or dsRNA binding (yellow nodes) were connected to different sets of interactors. Interestingly, comparison of the integrated sexual and aposporous networks suggested that apospory (Figure 4C) is accompanied by variation in the overall proportion of nucleic acid-binding proteins (blue nodes) versus proteins partially or completely lacking this ability (yellow nodes) which favoured the former class.

### 2.4. Spatial and Temporal Gene Expression Analyses in H. perforatum.

Time-course gene expression analysis was performed on pistils collected at different developmental stages spanning premeiosis (S1), meiosis (S2) and two terminal stages of gametogenesis (early: S3, late: S4) from both sexual and aposporous plant accessions. A principal component analysis [29] performed with the qPCR data separated early (S1 and S2) and late (S3 and S4) pistil developmental stages collected from sexual samples. The same PCA clustered sexual and aposporous samples according to the second component (Figure 3C). Notably, the clustering of samples along the first component placed the aposporous pistils in an intermediate position with respect to sexual early and late developmental timepoints. The only exception to this sample grouping was observed for apomictic sample S1 (corresponding to premeiotic pistils), which clustered in proximity to the sexual sample corresponding to gametogenesis (S3 and S4) (Figure 3C).

**Figure 5:**
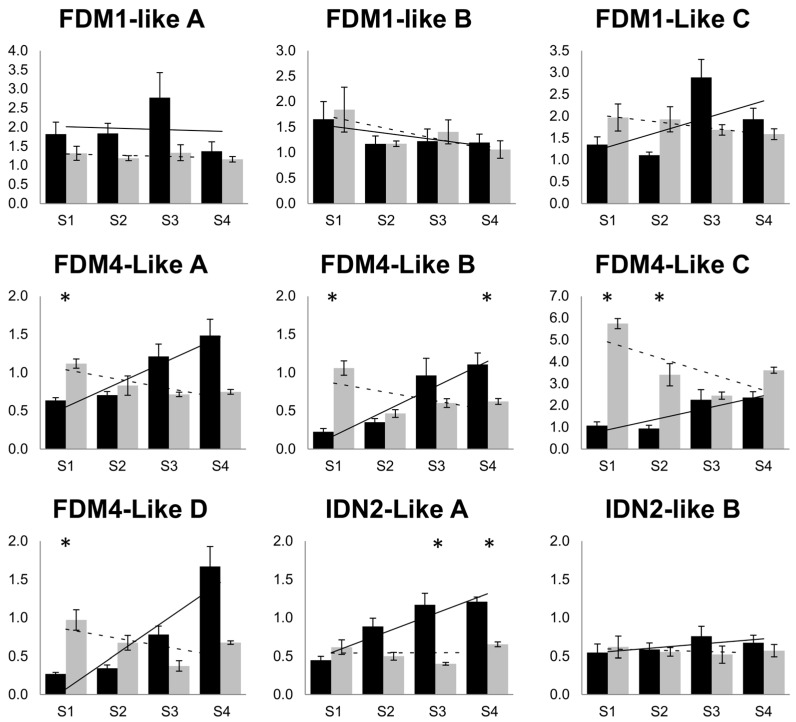
IDN2-like and FDM1,4-like gene expression analysis in pistils collected from sexual and aposporous plant accessions. Quantitative Real-Time qPCR were performed on four pistils developmental stages corresponding to pre-meiosis (S1), meiosis (S2), early and late gametogenesis (S3, S4, respectively). Black bars: expression recorded in sexual pistils; light grey bars: expression recorded in aposporous pistils. Relative expression values are plotted on the vertical axe. Error bars indicate the standard error observed among the five biological replicates. Significant differences (one-way ANOVA, *p*-value < 0.01) with the corresponding mock-treated controls are reported as: *.

At the single-gene level, no significant differences (ANOVA *p*-value cut off ≤ 0.05) in *FDM1-like* genes were recorded in sexual and aposporous pistils. However, the increase in the expression of *FDM1-like A* and *FDM1-like C* coinciding with the onset of gametogenesis (stage S3) observed in sexual pistils was not recorded in apomictic pistils collected at comparable developmental stages (Figure 5).

The results of the comparison of the FDM1-like C mean expression pattern in sexual and aposporous S1-S3 pistils were consistent with heterochrony in the expression pattern of this gene. For the *FDM4-like* genes, sexual samples were generally characterized by increased expression throughout pistil development, with higher expression levels detected in the two terminal stages corresponding to female gametogenesis (Figure 5).

Notably, pistils collected from aposporous plants displayed the opposite trend of expression for the same genes (i.e., *FDM4-like*). As a result, the *FDM4-like* genes exhibited significantly higher expression in aposporous pistils at stage S1 (premeiosis) and lower or comparable expression levels in terminal pistil developmental stages. The expression of *IDN2-like* genes showed alterative patterns in sexual pistils but not in aposporous ones. Indeed, while *IDN2-like B* displayed constitutive expression in both sample sets and a similar (i.e., steady) expression pattern was observed for *IDN2-like A* in aposporous pistils, the expression of the latter gene constantly increased throughout pistil development in sexual accessions (Figure 5).

### 2.5. Transcriptional Regulation of XH/XS Domain-containing Genes

To gain additional insight into the biological functions and transcriptional regulation of *HPIDN2-like* and *FDM1,4-like* genes, the 1.0 kb upstream sequence from the translation start was investigated for the presence of cis-regulatory elements. The computational annotation of promoter sequences identified 431 TFs belonging to 66 TF families (Tables S6 and S7). The most represented TF families that we detected were as follows: GATA tify, Dof, AT-Hook, ZF-HD, bZIP, bHLH and Myb/SANT_ARR-B. CIS-regulated elements associated with the responses to ethylene (ERFs, EILs, ERS), auxin (ARFs, MP), brassinosteroids (BEE1, BES1), abscisic acid (ABIs, ABFs, CBFs) and salicylic acid were also identified in *IDN2-like* and *FDM1,4-like* promoters. Most CIS-regulatory elements were detected only in subsets of the gene family, suggesting that multiple biotic and abiotic stimuli exhibit the potential to promote the transcription of the different *IDN2-like* and *FDM1,4-like* genes and that distinct regulatory factors might act in different plant contexts (Tables S6 and S7). The clustering of gene promoters according to the predicted TF binding sites grouped *IDN2-like B* and *FDM1-like A* together with FDM4-like C and far from *FDM4-like A/B* (Figure S4). Accordingly, B3/ARF CIS elements were only found in *IND2-like*, *FDM1-like A* and *FDM1-like C* genes (Tables S6 and S7). Moreover, promoter sequences recognized by B-type cytokinin response regulators were detected in the promoter sequences of *FDM1-like*, *FDM4-like C*/*D* and *IDN2-like B* but were absent in the promoters of *FDM4-like A*/*B* and *IND2-like A*. At the same time, CIS-regulatory elements recognized by CYTOKININ RESPONSE FACTORs (CRF1, CRF6) were detected in *IDN2-like* promoters but not in the promoter sequences of *FDM1,4-like* genes (Tables S6 and S7). Interestingly, the sequence recognized by the WUSHEL (WUS) homeobox gene controlling the stem cell pool was found in the promoters of *FDM1-like* A/B and *IDN2-like* genes. Notably, *FDM1-like A* and *IDN2-like B* but not the other promoter sequences, were predicted to bind WRKY28 (Tables S6 and S7).

**Figure 6:**
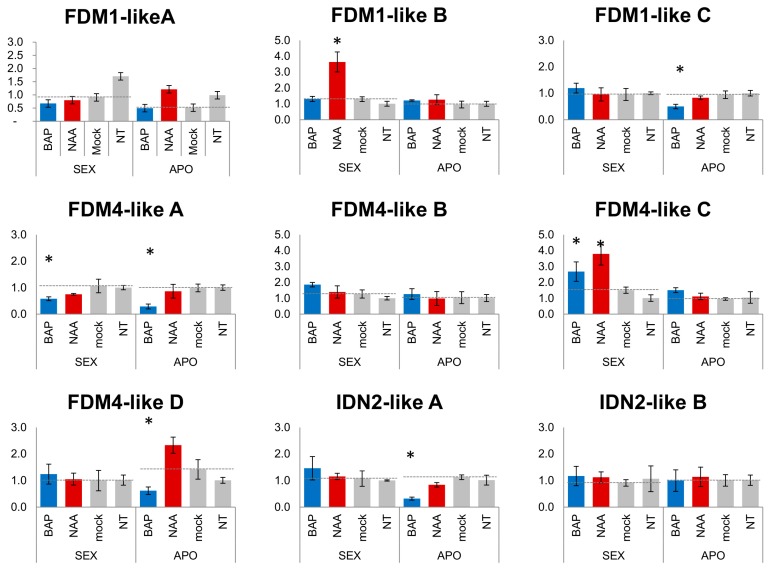
IDN2-like and FDM1,4-like transcript abundance in BAP and NAA treated pistils. Quantitative Real-Time qPCR were performed on pistils collected 1 h after the hormonal treatments. Treatments were performed on pistil developmental stages preceding gametogenesis (S1-S2), NT: untreated controls; mock: mock-treated controls. For each gene and considered phenotype (e.g., SEX vs. APO), relative expression values were referred to corresponding T0. Blue bars: expression recorded in BAP treated pistils (1h after the treatment); red bars: expression recorded in NAA treated pistils (1h after the treatment); light grey bars: expression recorded untreated pistils and mock-treated pistils. Relative expression values are plotted on the vertical axe. Error bars indicate the standard error observed among three biological replicates. Significant differences (one-way ANOVA, *p*-value < 0.01) with the corresponding mock-treated controls are reported as: *.

**Figure 7:**
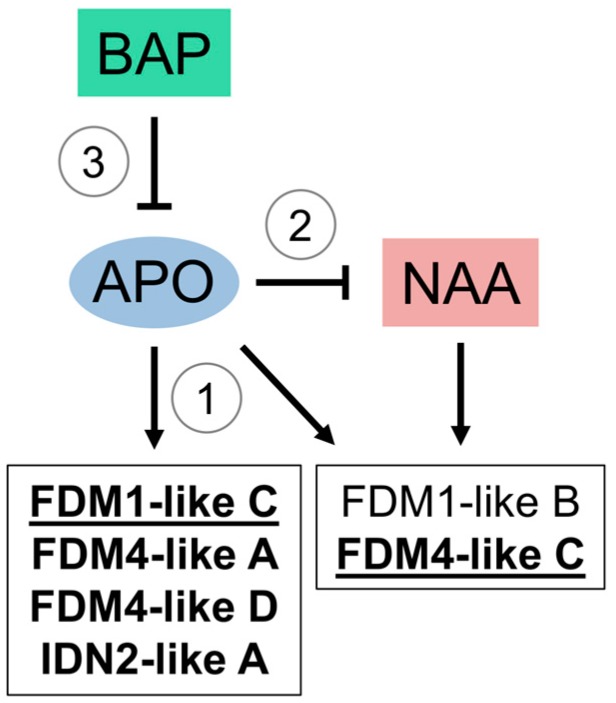
Interplay between hormonal responses and apospory in modulating FDM transcript abundance in *H. perforatum* pistils. ①: FDM1-like C and FDM4-like are overexpressed aposporous reproductive structures. ②: apospory antagonizes the transcriptional response of FDM1-like B and FDM4-like C to NAA treatments. ③: BAP represses the (positive) transcriptional response elicited by apospory. Genes differentially expressed in pistils are indicated with bold characters. Genes differentially expressed in ovules are underlined.

The identification of multiple CIS elements related to cytokinin and auxin responses in *HPIDN2-like* and *HPFDM1,4-like* promoters, together with the differential expression of several gene members in sexual and aposporous reproductive structures, raised the following two questions: (i) does an increased hormonal concentration affect the transcript abundance of these genes in reproductive structures (e.g., pistils)? and (ii) in the case of a transcriptional effect mediated by the two treatments, is this effect conserved in sexual and aposporous genotypes? To address these questions, we treated different cuttings originating from both sexual and aposporous plant accessions with synthetic analogues of the two hormones and measured the variation of *HPIDN2-like* and *HPFDM1,4-like* transcript abundance 1 h after the treatments (Figure 6). Several transcriptional responses were elicited by the two treatments, suggesting that the transcription of these gene members in pistils is, to so some extent, regulated by the two hormones (stage S1–S2). Remarkably, regarding the observed transcriptional changes, while treatment with NAA resulted in increased transcript abundance, the opposite effect (i.e., decreased transcript abundance) was most frequently associated with treatment with BAP (Figure 6). Indeed, while NAA significantly increased the transcripts of FDM1-like B and FDM4-like C in sexual pistils, no difference was recorded for the same genes in pistils collected from aposporous plants (Figure 6, Figure 7②). Moreover, the reduction of the transcript levels of *FDM1-like C*, *FDM4-like D*, *IDN2-like A* was only observed in pistils collected from aposporous plants (Figure 6, Figure 7③). However, a significant reduction of *FDM4-like A* transcript levels following BAP treatment was documented in both phenotypes (Figure 6). Notably, our RNAseq analysis showed that *FDM1-like C* and *FDM4-like C* were differentially expressed in aposporous ovules, suggesting that the modulation of their transcript levels in ovules could be related to local responses to auxin and cytokinin stimuli (Figure 6 and Figure 7).

## 3. Discussion

Ovule cell fate determination requires the expression of genes involved in posttranscriptional gene silencing (PTGS) mediated by sRNA [9,30,31,32,33]. Posttranscriptional gene silencing mediated by siRNAs might be associated with the RNA-directed *de novo* methylation of non-symmetrical CpG residues in targeted DNA loci and subsequent transcriptional gene silencing (TGS). The RNA-directed DNA methylation pathway in *A. thaliana* requires the expression of ATIDN2 and its interaction with at least two similar proteins known as FACTOR OF DNA METHYLATION 1 (ATFDM1) and FACTOR OF DNA METHYLATION 2 (ATFDM2) [11,12,13]. The protein domain composition, protein-protein interactions and co-expression network (Figure S5) suggest that 3 additional proteins, ATFDM3, ATFDM4 and ATFDM5, might also be involved in RNA-directed DNA methylation.

Although DNA methylation status in a subset of RdDM target loci is not affected in single *fdm3-1*, *fdm4-1* and *fdm5-1* knockout lines, double mutant analyses revealed that FDM3, FDM4 and FDM5 play redundant roles to FDM1 in RdDM [19]. The clustering of FDM-like genes based on their expression patterns across different plant contexts in Arabidopsis grouped *ATFDM4* apart from *ATIDN2*, *ATFDM1-2* and, to a lesser extent, *ATFDM.* In Arabidopsis, *ATFDM4* is the gene with the highest expression in shoot apical inflorescences, flowers at early developmental stages, carpels and ovaries. Within seeds and siliques, the expression of IDN2 and FDM1-3 peaks at seed developmental stages 4 and 7, while *ATFDM4* and *ATFDM5* display a decreasing trend of expression throughout seed development. Our phenotypic investigations revealed a 15% reduction of the seed set in *idn2* and *fdm1, fdm2, fdm3* and *fdm4* mutants. These phenotypic effects are in line with a sporophytic effect affecting the reproductive process, as observed for *AGO9* and other mutants affecting the RdDM pathway [31]. However, the higher frequency of aborted seeds observed in *fdm1* and *fdm2* mutants is consistent with partial differentiation of the activity of these genes with respect to FDM3 and FDM4. Accordingly, knockout of *IDN2* and the cluster I genes *FDM1, FDM2* and *FDM5* resulted in variability in embryo DNA content estimates. The observed reduction of the estimated genome size is not compatible with the parthenogenic development of both reduced and unreduced gametes. Additionally, apoptosis-associated chromatin degradation typically generates a much greater decrease in DNA stainability and comparable peaks in different mutant backgrounds. Furthermore, it is worth noting that despite the apparent genome size reduction, the seeds produced by *idn2*, *fdm1, fdm2* and *fdm5* plants can germinate and the generated plants set seeds, indicating that meiosis is mostly unaffected in single-gene knockout mutants, as otherwise expected in case of large genomic mutations involving 10% - 30% DNA loss. Estimates of embryo DNA content by flow cytometry rely on the accessibility of the DNA minor groove to DAPI and its relationship to chromatin structure [34]. Accordingly, the removal of the basic nuclear proteins, including histones, by acids or digestion with trypsin results in a marked increase in the accessibility of DNA to several dyes commonly used in flow cytometry, including DAPI [34]. It has been shown that IDN2-FDM complexes facilitate RdDM by altering nucleosome positioning through interactions with a subunit of the SWI/SNF chromatin remodelling complex [21]. Since nucleosome positioning might affect chromatin structure [35], it is possible that the reduction in DAPI-dependent embryo DNA contents observed in our mutants reflects a decrease in the accessibility of chromatin to DAPI, implying that nucleosome density is affected in *idn2* and Class I *FDM* mutants but not *fdm3* and *fdm4*.

As the acquisition of embryo sac (ES) cell fate from illegitimate cell precursors of the ovule nucellus is a key feature of aposporous apomixis in several species, including *H. perforatum* [3,4,5,6,7,8], comparative gene expression studies focused on *H. perforatum* reproductive structures might prove to be a powerful tool for dissecting genes involved in characteristic processes of the two reproductive strategies. Here, we report that two IDN2-like and 7 FDM-like proteins are encoded by the *Hypericum perforatum* genome. Our estimates of evolutionary divergence between sequences and phylogenetic analysis identified two *HPIDN2-like* genes (Cluster II), three *HPFDM1-like* genes (Cluster I) and four *HPFDM4-like* genes (Cluster III), which were named according to the Arabidopsis and Poplar gene nomenclature. As sequencing errors or inaccurate definition of intron-exon boundaries might lead to errors in the prediction of protein primary sequences [36] we focused our computational investigations on model species for which the genome sequence is available and by considering gene sequences marked as complete in the public databases. Despite the adoption of these stringent criteria for the selection of protein sequences, about 16% of the investigated protein sequences lacked the Zf-XS domain, while the XS and XH domain were missing in about 8.5% and 1.9% of investigated proteins and multiple Zf-XS domains were annotated in about 2.8% of considered sequences. Detailed investigations will be required to confirm the accuracy of the annotated sequences. However, we noticed that proteins lacking one or multiple domains were typically shorter than their putative orthologs. This might suggest that the observed variability in protein domains composition was associated to the lack of the corresponding sequence portions in the investigated gene sequences rather than misrecognition of protein domains within sequences. Both the HPFDM4-like A and HPFDM4-like C proteins lack the two N-terminal domains required for nucleic acid binding (i.e., Zf-XS and XS). However, the FDM4-like proteins display distinct and partially non-overlapping sets of predicted interactors, indicating functional differentiation among duplicated gene members. Interestingly, the lack of the central domain XS in our protein dataset was typically associated with the loss of the N-terminal Zf-XS domain and the only exception to this observation in our dataset is represented by the FDM4--like protein ONM30063 (Z. mays), which retains only the two terminal domains Zf-XS and XH. Interestingly, we noticed that within monocots and dicots, the higher number of protein sequences lacking one or multiple domains was observed in species with a greater number of gene members (e.g., *C. sinensis*, *G. max*, *H. perforatum*, *Zea mays*). At the same time, no deviations in the protein domain architecture were detected in plant species characterized by a lower number of predicted genes (e.g., *J. curcas*, *P. trichocarpa*, *R. communis*, *S. tuberosum*, *V. vinifera*). This high variability in protein domain composition, which was recorded in the moss *P. patens* together with several monocots and dicots, suggests that protein domain architecture in FDM-like proteins might be characterized relatively high plasticity. Compared to the sexually reproducing Arabidopsis and Poplar, the *H. perforatum* gene family has experienced expansion of the FDM4 clade. Among the considered species, only the polyploid *Glycine max* genome was annotated with four *FDM4-like* genes. By contrast, one to two FDM4-like genes were found in *M. esculenta*, *P. trichocarpa* and *R. communis,* which, together with *H. perforatum*, belong to the order Malpighiales. Based on the estimated evolutionary divergence between *FDM4-like* sequence pairs and gene order in the genomic sequence represented by ctg61006 (positions 220,754–241,969), we hypothesize that *FDM4-like B* and *FDM4-like C* originated from a duplication of a ~6 kb region from ctg61006, harbouring the two loci *FDM4-like D* – *FDM4-like A* or *vice versa*. Accordingly, the annotation of FDM4-like promoter sequences suggested alternative regulatory pathways, a prediction that is in line with the altered expression patterns observed in pistils treated with BAP and NAA. In this study, we integrated two independent datasets of transcriptomic data generated from a large number of tetraploid genotypes of both sexual and apomictic phenotypes and genomic data originating from one sexual diploid individual. Our computational investigations identified 18 transcript variants that were associated with nine genomic loci. The integration of RNAseq data with genome sequence assembly allowed the identification of multiple transcript variants (N: 1-4) sharing variable extents of sequence similarity with the reference gene sequences and characterized by alternative expression values. Three transcript variants were found to be differentially expressed in the ovule nucellus of aposporous plant accessions. These genes encode IDN2-like B, FDM1-like C and FDM4-like C. Furthermore, while downregulation of the highly expressed *IDN2-like B* gene in aposporous ovules was detected in both references (i.e., the sexual diploid genome and the transcriptome assembled *de novo* from both sexual and apomictic ovules), upregulation of two *FDM1-like C* and *FDM4-like C* transcript variants was only detected at the transcriptome level. Notably, sequence variability and expression levels appeared to be interconnected for these loci, as indicated by the differential expression of two highly divergent variants showing a short length of transcript/gene alignment.

Both the RNA-seq and qPCR gene expression data allowed consistent clustering of samples according to their phenotype, indicating that intraphenotype gene expression variation was lower than interphenotype variation. Remarkably, the adoption of highly specific primers to examine the expression of single gene members throughout pistil development highlighted extensive differences in the expression of FDM4-like genes in aposporous pistils. The apomictic accessions adopted for the hormonal treatments and gene expression analysis by qPCR were facultative apomictic (Table S8) characterized by the co-occurrence of both biological pathways within the same individua. Consequently, it is likely that transcriptional differences observed in pistils collected from sexual and apomictic accessions might have been underestimated due to the superposition of both reproductive pathways in facultative apomicts. Nevertheless, the expression of *FDM1-like C* and *FDM4-like* genes emphasized a clear dichotomy between the developmental stages preceding gametogenesis in sexual plants (i.e., S1, S2) and the following developmental stages (concomitant with gametogenesis). More specifically, the expression of the *FDM1-like C* and *FDM4-like* genes in aposporous samples is heterochronic, developmentally precocious and significantly higher at pistil developmental stages, which are concomitant with failure of meiosis and differentiation of the aposporous embryo sac precursors in aposporous genotypes. The heterochrony in the expression of these genes was such that a principal component analysis of qPCR expression data clustered premeiotic aposporous pistils (S1) with sexual pistils undergoing female gametogenesis (S3, S4). These expression patterns are consistent with the hypothesis that the transcriptional modulation of *HPFDM1-like C* and *HPFDM4-like* genes in *H. perforatum* reproductive structures is chronologically concomitant with the earliest biological differences detectable between sexual and aposporous ovules: apomeiosis and the acquisition of ES cell fate from illegitimate cell precursors [3,4,5,6,7,8]. Accordingly, the hybridization signals for *HPFDM1-like C* and *HPFDM4-like C* in *H. perforatum* reproductive structures were higher or were restricted to the ovule nucellus [24].

According to our current understanding of the molecular functions of ATIDN2, ATFDM1 and ATFDM2 [18,20], the activity of these proteins relies on protein-protein interactions involving the XH and coiled coil domains as well as DNA and RNA interactions involving the XS and ZF-XS domains. Our computational investigations of protein-protein interactions in *H. perforatum* predicted a complex network of potential interactions (edges: 16) between HPIDN2-like and HPFDM1,4-like proteins. Taken together, our RNAseq and qPCR gene expression patterns along with the predicted PPI and promoter annotations suggest that *IDN2-like A* and *IDN2-like B* are characterized by distinct transcriptional profiles, differential transcriptional regulation and alternative sets of FDM1-like and FDM4-like interactors. The proteins encoded by *IDN2-like B*, *FDM1-like C* and *FDM4-like C* were involved in most predicted interactions (n: 10/16). Remarkably, the integration of ovule gene expression data with PPIs suggested that the overall proportion of transcripts encoding DNA/RNA-interacting versus DNA/RNA-noninteracting proteins is remarkably different in sexual and aposporous ovules. Based on our current understanding of IDN2-FDM molecular functions, we hypothesize that (i) the activity of DNA/RNA-noninteracting proteins requires an association with DNA/RNA-interacting proteins; and (ii) the activity of DNA/RNA-noninteracting proteins does not require the binding of siRNA/lncRNA dsRNAs at RdDM-targeted loci. In both cases, considering that the association of DOMAINS REARRANGED METHYLTRANSFERASE2 (DRM2) with lncRNAs at RdDM-targeted loci in *A. thaliana* depends on both AGO4 and IDN2 [20], it seems reasonable to hypothesize that the altered proportions of DNA/RNA-interacting versus DNA/RNA-noninteracting proteins might influence the association and/or distribution of DRM2 at RdDM-targeted loci.

Regarding the transcriptional regulation of *HPIDN2-like* and *HPFDM1,4-like* genes, the annotation of CIS-regulatory elements in gene promoters was consistent with the activity of distinct, yet partially overlapping gene expression regulatory mechanisms. The identification of CIS elements recognized by WUSHEL (WUS) and/or SHOOT MERISTEMLESS (STM) in the promoters of *IDN2-like B* and *FDM4-like C* is in line with the expression recorded in Arabidopsis (Table S1, Figure S1) and *Hypericum,* as indicated by our RNAseq data and the hybridization signals in the nucellus of *H. perforatum* ovules (Figure S3). It is worth noting that other TRANS-acting factors involved in cell patterning or ovule development (INO, WRKY28, BP1, NACs and members of the KANADI family of putative transcription factors) were predicted to bind *HPIDN2-like B* and *HPFDM1-like* promoters.

The modulation of transcript abundance mediated by the BAP treatments was generally consistent with the annotated CIS-regulatory elements (e.g., type-B ARRs and CRFs, ARF). However, promoter annotations and expression studies of *HPFDM1-like A*, *HPFDM4-like A* and *HPIDN2-like B* suggested that transcript abundance might be modulated by complex regulatory mechanisms possibly involving multiple TRANS-acting factors with antagonistic activity and/or posttranscriptional gene expression regulation. Cell specification in ovules requires epigenetic regulation of gene expression by either RdDM or PTGS [9] and IDN2 and FDM-like are required for siRNA-mediated DNA methylation [11,12]. At the same time, cell proliferation and expansion in different plant contexts—including lateral organ development, apical meristems and ovules—are affected by cytokinins and auxins [37,38,39,40,41,42,43,44]. Therefore, the observed transcriptional responses of the RdDM-related genes *HPIDN2* and *HPFDM1,4-like* elicited by the hormonal treatments are particularly intriguing. The expression data for S1/S2 pistils (pregametogenesis), collected from both phenotypes and following BAP and NAA treatments, are consistent with the model reported in Figure 7, which summarizes the interplay between hormonal treatments and apospory in modulating FDM transcript abundance in reproductive structures (i.e., pistils).

Expression of the FDM1-like C and FDM4-like genes is elicited in ovules and/or higher order reproductive structures (i.e., pistils) of aposporous genotypes (Figure 7①). This elicitation is directly or indirectly promoted by one or more apospory controlling gene/s (defined herein as APO). This hypothesis is in line with the upregulation of these genes in pistils and the higher expression detected for FDM1-like C and FDM4-like C in aposporous ovules, as indicated by the RNAseq analyses of LCM microdissected cells. NAA increases the transcript levels of FDM1-like B and FDM4-like C in sexual but not aposporous pistils, leading to the conclusion that the transcriptional response of these genes is negatively regulated by APO (Figure 7②). However, increased expression of FDM4-like C was detected in both pistils and ovules collected from aposporous genotypes, indicating that additional as yet undetermined regulatory mechanisms might exist, at least for this gene. BAP decreases the transcript levels of FDM1-like C, FDM4-like A and FDM4-like D and IDN2-like A in aposporous but not sexual pistils. As the expression of FDM1-like C, FDM4-like A and FDM4-like D is promoted in aposporous genotypes, the repression of these transcripts only in aposporous samples is consistent with a model in which BAP directly or indirectly represses the (positive) transcriptional response elicited by APO (Figure 7③). The transcriptional effects exerted by the two treatments on FDM4-like C and IDN2-like A in sexual and aposporous pistils, respectively, might suggest synergic, rather than antagonistic responses. However, additional, not yet defined, factors might contribute to the observed transcriptional responses, including the crosstalk between the auxins and cytokinins [37], interplay with other hormones [45] and interaction with webs of local responses [37,46]. Although additional studies will be needed to clarify the causal relationship between apospory and cytokinin/auxin hormonal responses (e.g., one or more apospory-related genes modulating hormonal responses or *vice versa*), our expression data from BAP- and NAA-treated pistils demonstrated that the effects of the two synthetic hormones on *IDN2-like* and *FDM1,4-like* transcript levels are intimately connected to the reproductive phenotype. It is worth noting that recent research in the aposporous complex *Hieracium* spp. indicates that aposporus initials in this species are characterized by the overexpression of transcripts involved in the inhibition of cytokinin signalling, which could result in desensitization to the cytokinin signalling [47]. Our model in *H. perforatum* reveals two important aspects of the interplay between hormonal treatments and apospory in modulating FDM transcript abundance in reproductive structures: (i) the transcriptional responses on FDM-like genes in *H. perforatum* are determined by crosstalk between genetic determinants of apospory and response regulators elicited by NAA and BAP treatments; and (ii) the promotion of FDM transcript abundance mediated by the genetic determinants of apospory is repressed by the regulators of BAP responses.

In conclusion, aposporous *H. perforatum* reproductive structures with altered ES founder cell specification are characterized by differential and/or heterochronic expression of *IDN2-like* and *FDM1,4-like* gene. The transcript levels of *IDN2-like* and *FDM1,4-like* in pistils are affected by BAP and NAA treatments but the transcriptional responses elicited by the two synthetic hormones are dependent on the plant reproductive phenotype. This finding suggests that FDM transcript abundance in reproductive structures is modulated by a molecular network involving interplay between hormonal response regulators and apospory-controlling genes. Our findings contribute to elucidating the phylogeny, expression and regulation of *HPIDN2-like* and *HPFDM1,4-like* transcript abundance in *H. perforatum* reproductive structures and provide additional insight into transcriptional deviations and hormonal responses associated with the aposporous apomictic reproductive strategy in *H. perforatum.*

## 4. Materials and Methods

### 4.1. Bioinformatic analysis

#### 4.1.1. RNAseq in Ovule Microdissected Samples

The *H. perforatum* ovule transcriptome was assembled *de novo* and globally investigated in prior study [24]. Annotations of the *H. perforatum* ovule transcriptome was performed by using the stand-alone BLAST+ v2.7.1 [48] application. BLASTs were performed by using the BLASTX option (e-value cut-off = 1.0E-9) and by adopting the *A. thaliana* (TAIR10) and *P. trichocarpa* (V3) proteomes downloaded from the NCBI (http://www.ncbi.nlm.nih.gov/) (Table S5). Gene Expression analysis, mapping and sequence counts were performed with the software CLC Genomics Workbench V 7 (Qiagen), with default parameters and by using the de novo transcriptome as reference. Differentially expressed genes (DEGs) were identified by using the software empirical analysis of DGE [49] implemented in the CLC Genomics Workbench V 7 (Qiagen) and by adopting both FDR (*p*-value ≤ 0.05) and Holm *p*-value corrections (*p*-value ≤ 0.05). Holm corrected *p*-values were calculated with R (https://www.r-project.org/) and by using the p.adjust function.

Raw read counts were normalized by using the TMM normalization method implemented in the empirical analysis of DGE algorithm [49]. Sexual samples were adopted as references. To allow the comparison in the expression levels detected for different genes and driving from multiple sequencing libraries, the expression data reported in tables and HCL were referred to as RPKM [50].

*A. thaliana* gene expression data were retrieved from the BAR Expression Browser (http://bar.utoronto.ca/affydb/cgi-bin/affy_db_exprss_browser_in.cgi) [51]. Outputs display the average expression of replicate treatments relative to average of the appropriate control. Expression data were downloaded as graphical representation of log transformed clustered data. Heatmaps were generate with the software heatmapper (http://heatmapper.ca/pairwise/) [52]. Sample and gene clustering were generated by using the Pearson Correlation Coefficient and average linkage options.

#### 4.1.2. Annotation of Gene Sequences and Protein-Protein Interaction Prediction

Gene sequences were annotated by taking advantage of the *H. perforatum* genome sequence [24] and the available transcriptomic data [24,27] for this species. Genomic contigs most likely encoding for members of the IDN2/FDM-like gene family were initially identified by using the stand-alone BLAST+ v2.7.1 application [48] and by adopting the *H. perforatum IDN2* and *FDM-like* transcripts as queries (e-value cut-off: 1.0E-20). Contigs sharing some similarity with *IDN2* and *FDM-like* transcripts were selected and annotated in further detail. For each putative gene locus, the sequence portion providing the aforementioned alignments was extended upstream to find the putative start-codon (ATG) [53,54] and downstream to find the putative stop-codon (TAG, TAA, TGA) [55]. Coding sequences were predicted from the alignment of transcript and gene sequences. CDS predictions were manually verified by BLASTX over the nr database (http://www.ncbi.nlm.nih.gov/). Functional domains were annotated using Conserved Domain Database (CDD: https://www.ncbi.nlm.nih.gov/Structure/cdd/wrpsb.cgi) in order to verify the presence of peculiar domains in whole dataset [56,57]

For Genome-based expression analysis, mapping and sequence counts were performed with the software CLC Genomics Workbench V 7 (Qiagen), with default parameters. Sequencing reads were mapped on gene regions ±500 bp to include UTRs. Differentially expressed genes (DEGs) were identified by using the software empirical analysis of DGE [49] implemented in the CLC Genomics Workbench V 7 (Qiagen) and by adopting both FDR (*p*-value ≤ 0.05) and Holm *p*-value corrections (*p*-value ≤ 0.05). For the differential expression analysis, raw read counts were normalized by using the TMM normalization method implemented in the empirical analysis of DGE algorithm [49].Sexual samples were adopted as references. To allow the comparison in the expression levels detected for different genes and driving from multiple sequencing libraries, the expression data reported in tables and HCL were referred to as RPKM [50].

The heat map reporting on the expression of *IDN2* and *FDM-like* transcripts in ovules collected from sexual and aposporous accessions was performed by using the Hierarchical Clustering (HCL) algorithm implemented on T-MeV v4.9.0 software (http://mev.tm4.org) [58]. Gene transcripts and samples were clustered by using Manhattan distances and the average linkage clustering option.

CIS-regulatory elements were predicted by analysing the 1.0kb upstream sequence from the translation start with the software PlantPAN3 (http://plantpan.itps.ncku.edu.tw/promoter.php) [59]. The clustering of promoter sequences based on annotated CIS-regulatory elements was performed with the software plotted by using the software heatmapper (http://heatmapper.ca/pairwise/) [52]. As input file, we adopted a binary matrix reporting the presence (1) versus absence (0) of each predicted CIS-elements in the investigated promoter sequences. The heatmap was generated by using Manhattan distances and average linkage clustering options.

Protein-protein interactions were predicted by using the software PSOPIA (https://mizuguchilab.org/PSOPIA/) [60]. Pairwise comparisons were performed by using the *A. thaliana*, *P. trichocarpa* and *H. perforatum* predicted protein sequences. Sseq scores were plotted by using the software heatmapper (http://heatmapper.ca/pairwise/) [52]. The correlation matrix was calculated by using the Pearson Correlation Coefficient. The protein interaction network was generated with cytoscape 3.7.1 (https://cytoscape.org/). Network nodes represent the annotated *H. perforatum* proteins, while edges represent interactions predicted with PSOPIA. Only PPIs with scores ≥ 0.419 were included in the network. Nodes size were set based on mean gene expression values in sexual and aposporous libraries (Table S4).

#### 4.1.3. Phylogenetic Analysis

IND2 and FDM-like sequences (Table S5) were downloaded from ncbi (https://www.ncbi.nlm.nih.gov/protein/). Sequences were aligned by using the MAFFT algorithm implemented in TranslatorX (http://translatorx.co.uk/) [61] with default options. Both amino acid and nucleotide alignments were used in phylogenetic analyses through the Maximum Likelihood approach, carried out using IqTree v1.6.7 software [62]. Analysis on both datasets were carried out with best-fit models. Analysis on protein aligned dataset was performed with JTT+F+I+G4 model, while analysis on nucleotide dataset was performed with TIM2+F+I+G4 model. Both best-fit models were calculated using ModelFinder Plus [63] implemented in Iqtree software. Fifty independent tree searches were carried out, in order to avoid falling into local minimum events [62,64]. Topologies of each independent run were compared with the Robinson-Foulds [65] distance implemented in IqTree. Ultrafast Bootstrap support [66], for 10^6^ replicates, was calculate for each topology generated. Trees were rooted on *Physcomitrella patens.*

### 4.2. Plants Materials

*Arabidopsis thaliana* knockout lines N575378 (*fdm1*), N813508 (*fdm2*), N520841 (*fdm3*), N508738 (*fdm4*), N685934 (*fdm5*), N652144 (IDN2) were retrieved from the Nottingham Arabidopsis Stock Centre (NASC). *A. thaliana* Columbia (Col-0) accession was used in all experiments as plant materials and controls (WT). Surface-sterilization of wild-type and silenced line seeds was performed by immersion in 70% ethanol for 2 min followed by 10 min in a 30% (v/v) commercial bleach solution and finally 5 rinses with sterile water. Sterile seeds were then plated in Petri dishes (Ø 10 cm) containing 0.5× Murashige and Skoog [67] salts, 0.5% (*w*/*v*) sucrose (Sigma), 1% agargel (Sigma) and were left for 3 days at 4 °C in a dark chamber to synchronize their germination. Kanamicin (50 mg/mL) was added for selecting silenced lines and plates were incubated at 22 °C following growth conditions reported by Harrison, et al. [68] for a fast selection protocol. The genotyping of KanR plants was performed by PCR, by using primer combinations—LP/RP and LBb1.3 obtained from iSect Primers (http://signal.salk.edu/tdnaprimers.2.html) for each mutant line, as indicated by NASC [69]. Three to five independent homozygous plants were used in all experiments. Seedlings were then transplanted and grown under the same environmental conditions (12: 12 h light: dark at 23 °C).

Tetraploid (2*n* = 4*x* = 32) *Hypericum perforatum* accessions adopted in this study are reported on table S8. Plants were grown on soil inside a greenhouse placed in Azienda Agraria Sperimentale “Lucio Toniolo,” Legnaro (PD, Italy). Phenotyping of *H. perforatum* accessions for their main reproductive mode has been performed by flow cytometric screening of single seeds as reported by Matzk, et al. [70]. The average number of analysed seeds per accession was 28, ranging from 16 to 49 (Table S8). Briefly, a single seed was inserted into an Eppendorf tube with a metal ball bearing and covered with 100 μL of nuclei extraction buffer (CyStain^®^ UV precise T kit by Sysmex). The seeds were ground in a TissueLyser II from Qiagen for 30 s. Following homogenization, 100 μL of nuclei extraction buffer were added to the sample. At this point, preparations were let chill for five minutes. After that, samples were auditioned with 800 μL of staining buffer (CyStain^®^ UV precise T kit by Sysmex) and stored for some minutes in the dark. The total volume was transferred in a cytometric tube that has a 30 μL mesh width nylon tissue filter on the top. Flow cytometric runs were performed with a Cyflow Ploidy Analyzer (Sysmex-Partec). The obtained profiles were than analysed by using the FCS express 5 Flow research software (Sysmex-Partec). The same experimental procedure was adapted for the flow cytometric analyses of WT and knockout Arabidopsis seeds (N. seeds/line: 16).

### 4.3. Expression Analysis by Real-Time qPCRs and in situ Hybridization Assays

Plant materials were selected according to the genetic and cyto-histological bases of apospory recently described for *H. perforatum* [4,71]. The reproductive mode of all *H. perforatum* accessions was estimated by the flow cytometric screening of 48 single seeds as indicated by Matzk, et al. [70]. Samples were collected separately from a minimum of five plant accessions (Table S8). Total RNA was extracted from collected pistils using the Spectrum™ Plant Total RNA Kit (Sigma-Aldrich), by following the protocol provided by the manufacturer. The eventual contamination of genomic DNA was avoided by using optional DNase I (Sigma-Aldrich) treatment. The abundance and pureness of RNAs were assessed using a NanoDrop 2000c UV-Vis spectrophotometer (Thermo Scientific, Pittsburgh, PA). cDNA synthesis was performed using the RevertAid First Strand cDNA Synthesis Kit (Thermo Scientific), following the instructions of the supplier. Primers used in the Real-Time RT-PCR experiments are reported in Table S9. Expression analyses were performed using StepOne thermal cyclers (Applied Biosystems) equipped with 96-well plate systems, respectively, with SYBR green PCR Master Mix reagent (Applied Biosystems). The amplification efficiency was calculated from raw data using OneStep Analysis software (Life Technologies). Amplification performance expressed as fold change was calculated with the ΔΔCt method using HpTIP4 as a housekeeping gene [72]. Error bars indicate the standard error observed among the five biological replicates.

### 4.4. Hormonal Treatments

Hormonal treatments were carried out on cuttings generated from 3 sexual and 3 aposporous apomictic plants (Table S8). A minimum number of 4 cuttings were performed each donor plant. Briefly, young and vigorous branches, originated from axillary buds of donor plants (Table S8), were excised during the vegetative stage and the bases were immediately put in a commercial powder of rooting hormone (Rigenal P - Var 5, CIFO) and then planted in a pot with sand, which had been previously hydrated with abundant water. Cutting were then placed into a humidity chamber, away from direct sun light in Azienda Agraria Sperimentale “Lucio Toniolo” for 4 weeks [73]. Cuttings were kept moist during all time and after 4 weeks we checked for roots by tugging gently and testing the resistance of cuttings. Once they have developed roots, they were removed from the humidity chamber and transferred into new pots. Cutting were transferred in greenhouses and allowed to flower.

**Figure 8:**
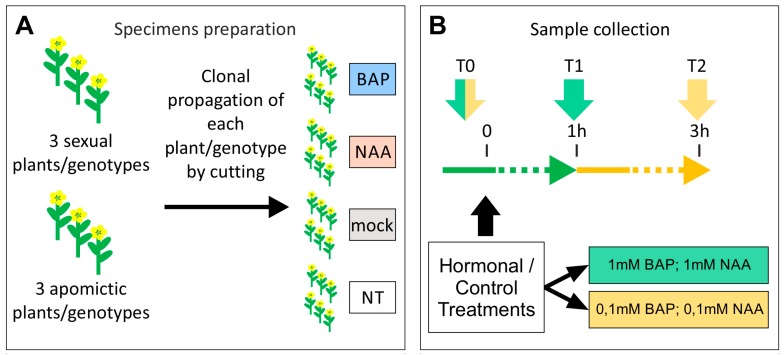
Experimental design adopted for the hormonal treatments. (**A**): experimental procedure adopted for the clonal propagation of *H. perforatum* plants/genotypes. Clonal propagation of 3 sexual and 3 apomictic plants/genotypes has been performed by cutting. A minimum number of four cuttings/plant was generated (for a total 24 plants). This strategy allowed to apply multiple hormonal and control treatments to single genotypes, by avoiding overlapping hormonal responses potentially deriving from the execution of multiple treatments on the same plant. (**B**): BAP and NAA hormonal treatments. Trial experiments were performed by using 2 hormone concentrations (0.1mM and 1mM). Samples were collected immediately before the treatments (T0), 1 h after the treatments (T1) and 3 h after the treatments (T2). The data discussed in the present manuscript were originated by using BAP and NAA to final concentration of 1mM (green box) and by collecting the samples at the two timepoints: T0 and T1 (green arrows).

6-Benzylaminopurine (BAP) and 1-Naphthaleneacetic acid (NAA) were obtained from Sigma-Aldrich. BAP and NAA were dissolved in 0.1 n NaOH to the concentration of 100mM and then diluted to a final concentration of 0.1mM and 1mM [40,74] in distilled water added with 0.05% Tween 20. For the BAP and NAA treatments, flower buds were treated once by floral dip. Hormonal and control treatments were performed on S1-S2 flower developmental stages. Trial experiments were performed by using BAP and NAA with both concentrations, in separate experiments and by collecting samples immediately before the hormones treatments (T0), 1 h after the treatments (T1) and 3 h after the treatments (T2) (Figure 8). The transcriptional effect exerted by the two hormones at both concentrations and timepoints was tested by qPCR on three reference genes (FDM1-like B and two A-type ARRs, data not shown). Gene expression data reported in this manuscript were originated by using three sexual and three apomictic plants/genotypes (biological replicates). The data discussed in the present manuscript were originated by using BAP and NAA to final concentration of 1mM and by collecting the samples at the two timepoints: T0 and T1. RNA extraction, cDNA synthesis and Real-Time qPCRs were carried out as described before. Amplification performance expressed as fold change was calculated with the ΔΔCt method using HpTIP4 as a housekeeping gene [72]. For each control and treated sample, the corresponding T0 was adopted as reference. Error bars indicate the standard error observed among the five biological replicates. Significant expression differences with the corresponding mock-treated controls were calculated with the method one-way ANOVA (*p*-value < 0.01).

## Figures and Tables

**Table 1:** Annotation and expression of genes encoding for XH/XS-domain containing proteins in *H. perforatum*. For each contig ID, the table reports on the contig length, gene coordinates (estimated from the start – stop codons), the orientation of predicted gene sequences, the predicted gene size and the associated gene name. For each contig ID, the table also reports on the aligned transcript variants, the percentage of identical residues in the alignment and the percentage of transcript sequence aligned to the corresponding gene. Metrics concerning the RNAseq expression values were generated by using the transcriptome assemble de novo as reference. For each transcript ID, the table reports on the mean expression value in sexual (SEX RPKM) and aposporous (APO RPKM) samples and corresponding Holm *p*-value correction and FDR *p*-value. * indicates corrected *p*-values lower than 5.0E-2.

Contig ID (Size)	Start–End (Orientation)	Gene Size (bp)	Gene Name	Transcript ID (Ovule RNAseq)	Identical Sites (%)	Alignment Length (%)	SEX RPKM	APO RPKM	Holm *p*-Value	FDR *p*-Value
61981 (562959 bp)	167,628–170,545 (−)	2917	FDM1-like A	L1163_T42_51	99.8	96.9	2441.7	2325.3	1E+00	1E+00
				L2_T426266_835163	99.1	92.8	334.7	160.3	1E+00	9E−01
	142,406–145,582 (−)	3176	FDM1-like B	L2_T405103_835163	99.8	62.9	1718.3	1123.7	1E+00	9E−01
54789 (39545 bp)	9–2763 (−)	2755	FDM1-like C	L2_T208885_835163	98.8	92.9	328.3	237.0	1E+00	1E+00
				L2_T208875_835163	95.5	89.0	3.7	2.3	1E+00	1E+00
				L2_T405131_835163	99.6	82.6	730.3	669.7	1E+00	1E+00
				L2_T405120_835163	100.0	41.7	0.3	90.3	3E-04 *	1E−06 *
61606 (370668 bp)	239,715–241,969 (+)	2255	FDM4-like A	L2_T485123_835163	84.8	29.3	1435.7	1350.3	1E+00	1E+00
	220754–224,058 (+)	3305	FDM4-like B	L2_T484442_835163	97.1	92.2	262.7	133.3	1E+00	9E-01
				L2_T484959_835163	96.3	61.3	5.7	4.3	1E+00	1E+00
	225,378–232,682 (+)	7304	FDM4-like C	L2_T485004_835163	94.3	90.1	840.7	1744.0	1E+00	3E-01
				L2_T484402_835163	89.3	61.0	0.7	223.3	1E-05 *	9E−08 *
	235,763–238,325 (+)	2563	FDM4-like D	L2_T485003_835163	85.5	42.7	4267.7	5010.3	1E+00	1E+00
60014 (81345 bp)	24,915–28,181 (−)	3267	IDN2-like A	L2_T689185_835163	99.4	97.7	7.3	5.3	1E+00	1E+00
				L2_T689193_835163	99.3	98.1	921.0	1111.0	1E+00	1E+00
61764 (56897 bp)	22,007–24,927 (+)	2921	IDN2-like B	L5704_T2_3	99.6	100.4	663.0	225.3	1E+00	3E−01
				L2_T781699_835163	99.1	88.5	80.7	13.0	1E+00	3E−01
				L13153_T20_20	96.1	88.6	3456.7	651.7	3E−01	7E−04 *

**Table 2:** IDN2 and FDM-like protein-protein interaction scores. The table reports on the two sequence-based PPI score Sseq and Sall. Interactions were predicted with PSOPIA and filtered by using the PPI score estimated for the experimentally determined *A. thaliana* IDN2/FDM1 heterodimer (PPIs scores: 0.419) as cut-off value. Homomeric interactions are displayed in the diagonal.

Heading	IDN2-like A	IDN2-like B	FDM1-like A	FDM1-like B	FDM1-like C	FDM4-like A	FDM4-like B	FDM4-like C	FDM4-like D
IDN2-like A	ns/ns								
IDN2-like B	ns/ns	ns/ns							
FDM1-like A	ns/ns	0.419/0.419	ns/ns						
FDM1-like B	ns/ns	ns/ns	ns/ns	0.419/0.714					
FDM1-like C	0.419/ns	ns/ns	ns/ns	0.419/0.714	0.419/0.714				
FDM4-like A	ns/ns	ns/ns	ns/ns	ns/ns	ns/ns	ns/ns			
FDM4-like B	ns/ns	0.419/0.419	ns/ns	ns/0.7686	ns/0.769	0.419/0.419	ns/0.769		
FDM4-like C	ns/ns	ns/ns	ns/ns	0.419/0.714	ns/0.769	ns/ns	ns/0.789	ns/0.789	
FDM4-like D	0.419/0.419	0.419/0.419	ns/ns	ns/ns	ns/ns	ns/ns	ns/ns	ns/ns	ns/ns

## Data Availability

Raw sequences files were made available for download from SRA with the following accession numbers SAMN10880815,S 880814, SAMN10880813, SAMN10880812, SAMN10880811, SAMN10880810. The Transcriptome Shotgun Assembly project has been deposited at DDBJ/EMBL/GenBank under the accession GHFN00000000. The version described in this paper is the first version, GHFN01000000. The *H. perforatum* genome was submitted as WGS submission with the accession number SOPF00000000. The expression data discussed in this publication have been deposited in NCBI’s Gene Expression Omnibus [75] and are accessible through GEO Series accession number GSE128923 (https://www.ncbi.nlm.nih.gov/geo/query/acc.cgi?acc=GSE128923). The *H. perforatum* IDN2-like and FDM-like gene sequences were deposited in GenBank with accession numbers: MK883483- MK883491.

## Supplementary Materials

The following are available online at https://www.mdpi.com/2223-7747/8/6/158/s1.
