# Supplementary material for: Annotation and Expression of IDN2-like and FDM-like Genes in Sexual and Aposporous Hypericum perforatum L. accessions
Source: Plants (Basel). 2019 Jun 7;8(6):158. doi: 10.3390/plants8060158 (PMC6631971; doi:10.3390/plants8060158)
Supplement: Supplementary file 1 [file plants-08-00158-s001.zip › plants-512978-supple/Supplementary.pdf]

Article

Annotation and Expression of IDN2-like and FDM-like Genes in Sexual and Aposporous *Hypericum perforatum* L. accessions

Andrea Basso, Gianni Barcaccia and Giulio Galla *

Laboratory of Genetics and Genomics, DAFNAE, University of Padova, Campus of Agripolis, Viale dell' Università, 1635020 Legnaro, Italy; andrea.basso@unipd.it (A.B.); gianni.barcaccia@unipd.it (G.B.)

* Correspondence: giulio.galla@unipd.it

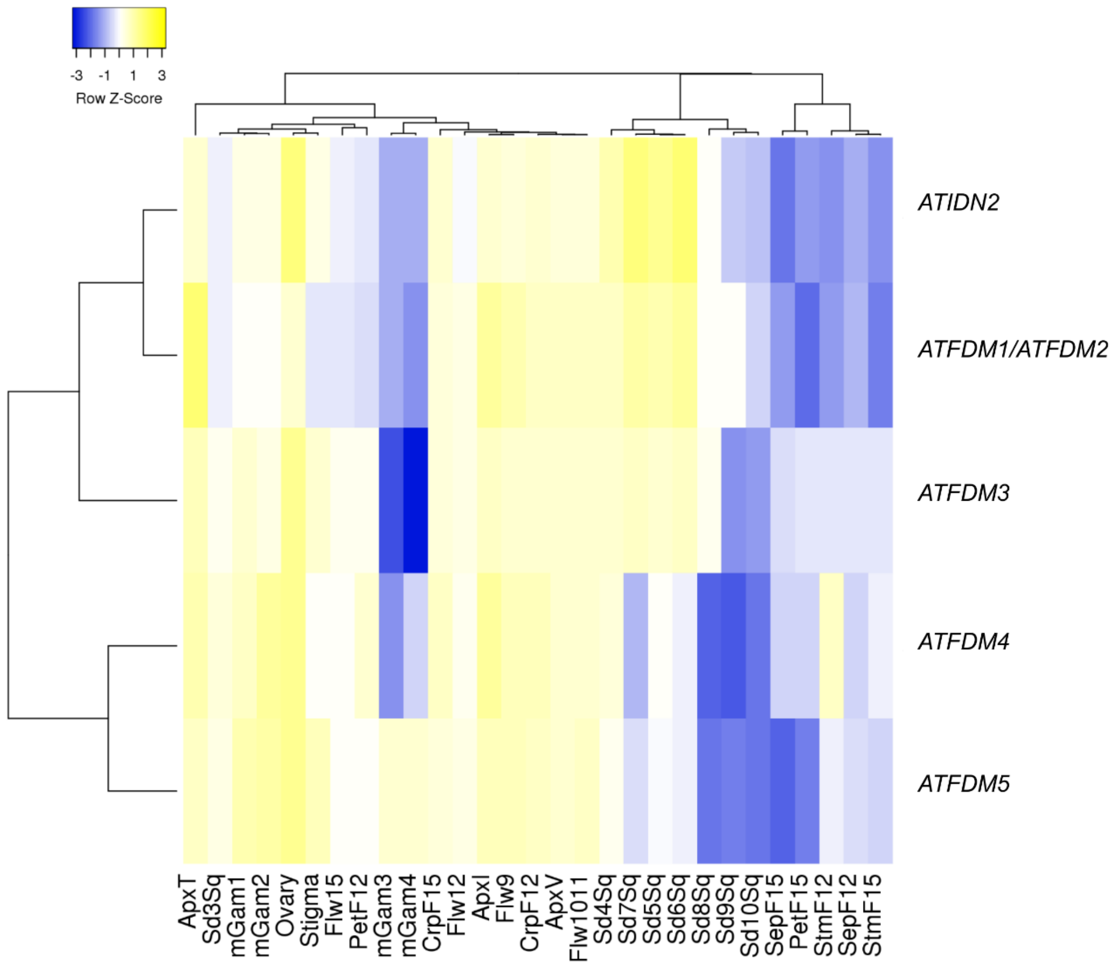

Figure 1. IDN2 and FDM1-5 gene expression in Arabidopsis. Heat map showing the expression of IDN2 and FDM1-5 genes in Arabidopsis lowers flower parts, seeds, male gametes and meristems. Gene expression data were retrieved as the average expression of replicate treatments relative to average of the appropriate control from (http://bar.utoronto.ca/affydb/cgi-bin/affy_db_exprss_browser_in.cgi). Sample and genes were clustered by using the Pearson Correlation Coefficient and average linkage options.

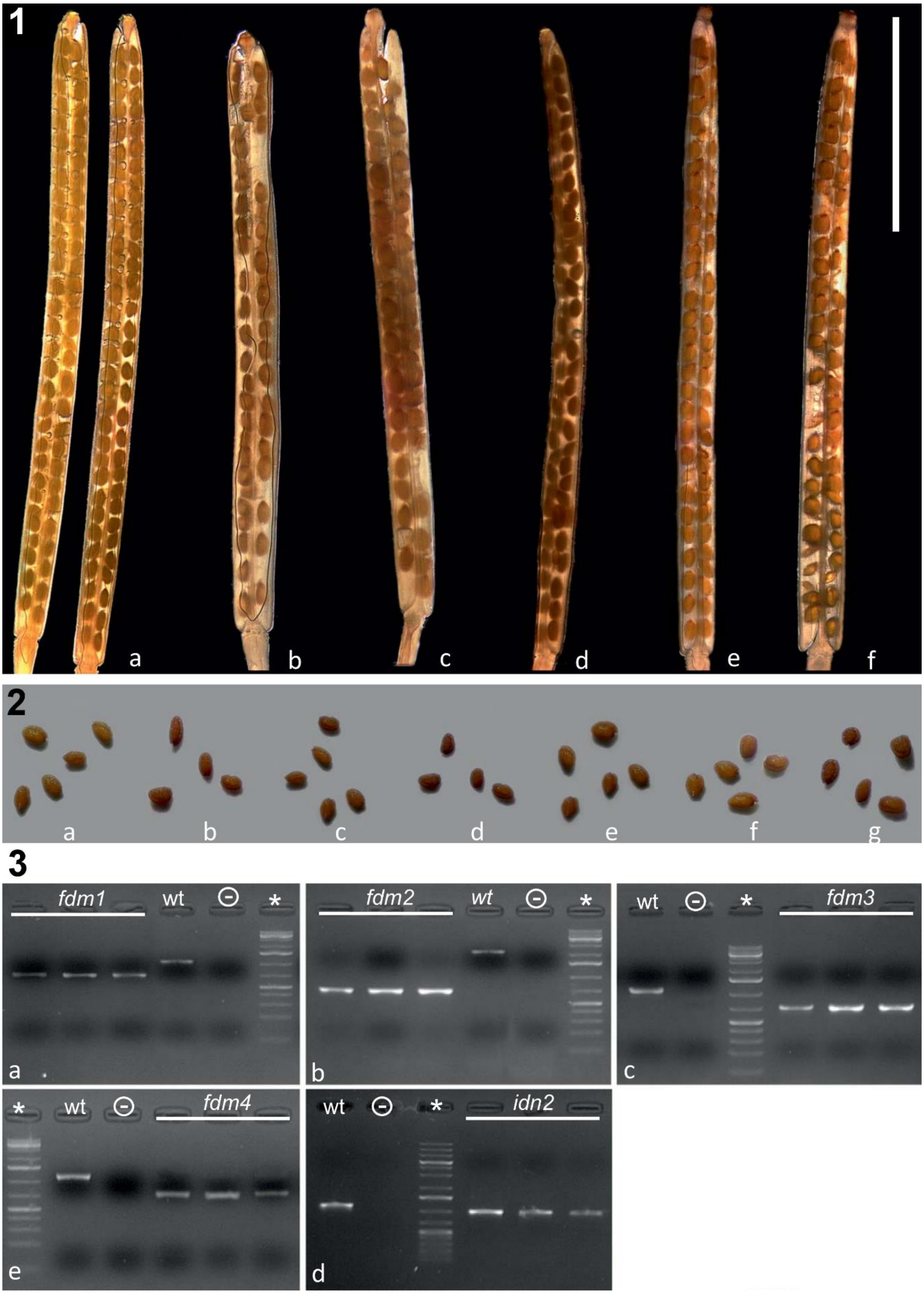

Figure 2. Panel 1: representative images of Arabidopsis thaliana wild type and mutant siliques. a: wild type; b: fdm1; c: fdm2; d: fdm3; e: fdm4; f: idn2; scale bar= 5mm. Panel 2: representative images of Arabidopsis thaliana wild type and mutant seeds: a: wild type; b: fdm1; c: fdm2; d: fdm3; e: fdm4; f: fdm5; g: idn2. Panel 3. Genotyping of KanR plants by PCR and by using primer combinations: LP/RP and LBb1.3. a: fdm1; b: fdm2; c: fdm3; d: fdm4; e: idn2; -: negative control; wt: wild type; *: 1kb GeneRuler 1 kb Plus DNA Ladder (Thermo Fisher Scientific).

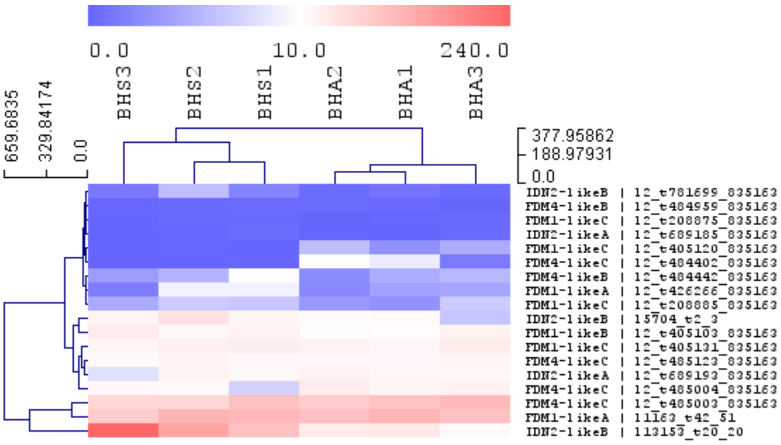

Figure 3. Expression of H. perforatum IDN2-like and FDM1,4-like genes. The Heat map was generated with the expression of IDN2-like and FDM1,4-like genes as assessed by RNAseq and by using the transcriptome assembled de novo from sexual and aposporous RNAseq data. The HCL was performed by using Manhattan distances and average linkage clustering. BHS1-3: sexual samples; BHA1-3: aposporous apomictic samples. Gene expression is reported as RPKM. Blue: low expression levels; Red: high expression levels.

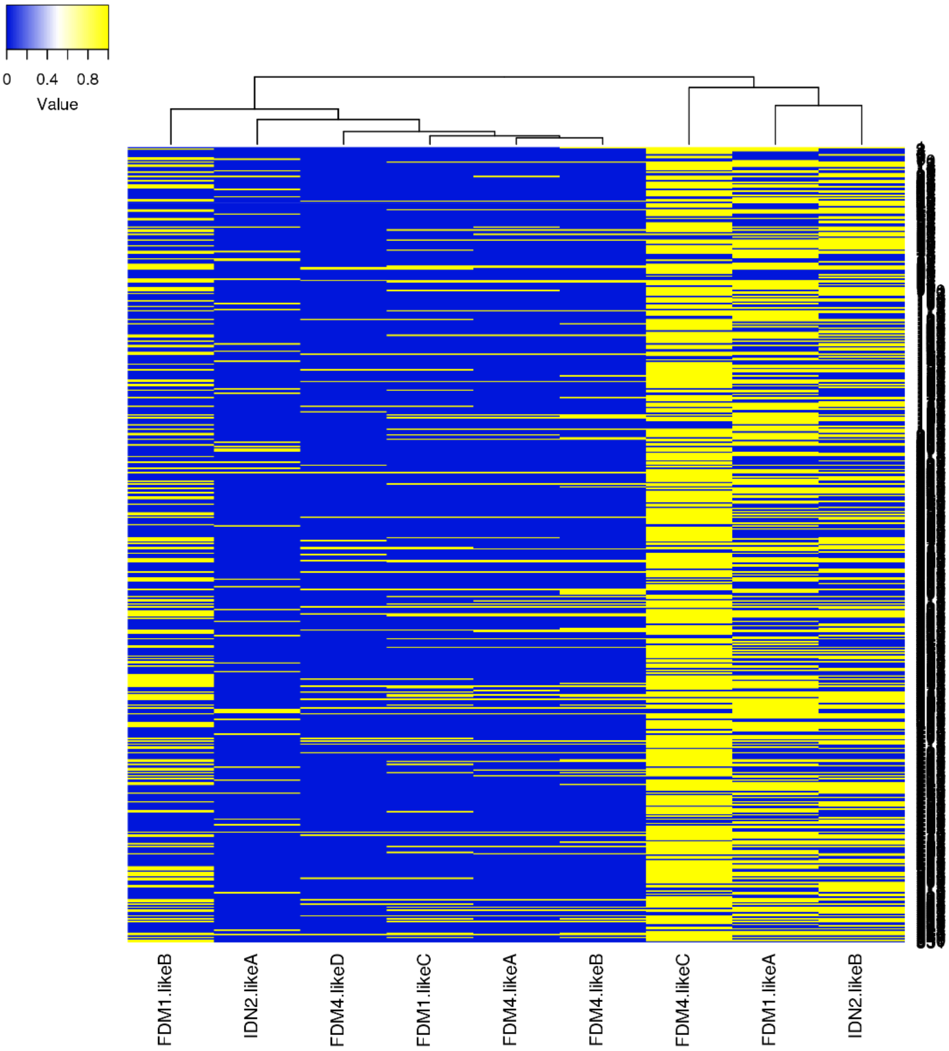

Figure S4. Clustering of IDN2-like ad FDM1,4-like promoter sequences based on predicted CIS-regulative elements. The heatmap was generated from a binary matrix reporting the presence (1) vs absence (0) of each predicted CIS-elements in the investigated promoter sequences. Gene promoters were clustered by using Manhattan distances and average linkage clustering options.

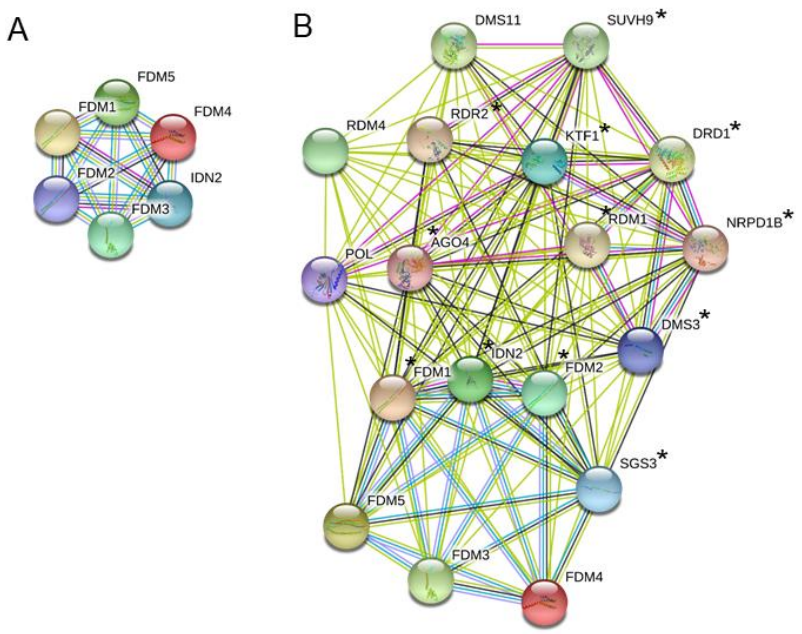

Figure 5. Known and predicted interactions between Arabidopsis IDN2 and FDM1-5 proteins. A: IDN2, FDM1-5 proteins interaction network. Nodes represent A. thaliana IDN2, FDM1-5 proteins, while edges represent predicted or experimentally determined protein-protein interactions. The average local clustering coefficient is 0.876. The PPI enrichment p-value is ≤ 1.0e-16. B: Known and predicted interactors of Arabidopsis IDN2 and FDM1-5 proteins. B: The network was generated by including IDN2, FDM1-5 proteins and the of up to 12 predicted interactors. Interactions colour legend: Blue: curated databases; pink: experimentally determined; black: co-expression analysis; Yellow: text mining. The PPI enrichment p-value is ≤ 1.0e-16. Proteins known to be involved in RdDM are marked with * .
